# Chest ultrasound vs. Radiograph for pneumothorax diagnosis performed by emergency healthcare workers in the emergency department: a systematic review and meta-analysis

**DOI:** 10.1186/s13089-025-00441-5

**Published:** 2025-07-31

**Authors:** Jean-Baptiste Bouillon-Minois, Coline Burlet, Resa E. Lewiss, Reza Bagheri, Christophe Perrier, Jeannot Schmidt, Frédéric Dutheil

**Affiliations:** 1 CHU Clermont-Ferrand, Emergency Department, CNRS, LaPSCo, Physiological and Psychosocial Stress, Université Clermont Auvergne, 58, Rue Montalembert, 63000 Clermont-Ferrand, France; 2 Emergency Department, CHU Clermont–Ferrand, 63000 Clermont-Ferrand, France; 3 Department of Emergency Medicine, The University of Alabama at Birmingham, Birmingham, AL USA; 4 Department of Exercise Physiology, University of Isfahan, Isfahan, 81746-73441 Iran; 5 CNRS, LaPSCo, Physiological and Psychosocial Stress, CHU Clermont–Ferrand, Occupational and Environmental Medicine, WittyFit, Université Clermont Auvergne, 63000 Clermont–Ferrand, France

**Keywords:** Bedside ultrasound, Chest ultrasound, Chest x-ray, Pneumothorax, Emergency department

## Abstract

**Background:**

The efficacy of bedside chest ultrasonography for the detection and diagnosis of pneumothorax is under debate. We aimed to compare Emergency Healthcare Workers performed chest ultrasonography with chest X-ray in the detection and diagnosis of pneumothorax in the emergency department.

**Methods:**

We queried PubMed, Cochrane, ScienceDirect, Web of Science and ClinicalTrials.gov databases from 2000 through January 2024. We included all studies (both retrospective and prospective) that compared the diagnostic performance of chest ultrasonography with chest radiography, using chest computed tomography as the gold standard. Participants are patients consulting in the emergency department and physician that performed the chest ultrasound was an Emergency Healthcare Workers. Studies reporting the sensitivity and specificity for both chest ultrasonography and chest X-ray met inclusion criteria. We applied a random effects meta-analysis methodology. We then performed a meta-regression analysis to search for influencing variables such as technical parameters of echograph, patients and pneumothorax.

**Main results:**

15 studies totaling 3,171 patients were analyzed. 71% of patients were male with a mean age of 40.2 years. The mean prevalence of pneumothorax was 27.6% (95 CI 20.9 to 34.3). Chest ultrasonography had higher sensitivity (79.4%, 68.2 to 90.7) compared to chest X-ray (48.1%, 36.8 to 59.4), and a greater negative predictive value (chest ultrasonography = 94.3%, 91.2 to 97.3, and chest X-ray = 87.9%, 84.1 to 91.6). There was no statistical difference in specificity between the two modalities: chest ultrasonography 99.5%, 99 to 100 and chest X-ray 99.8%, 99.4 to 100) or in positive predictive value (chest ultrasonography 94.2%, 90.5 to 97.9 vs chest X-ray 96.7%,92 to 100). Characteristics of echograph or pneumothorax and patients sociodemographic did not influence results.

**Conclusion:**

In this systematic review and meta-analysis, chest ultrasonography performed by Emergency Healthcare Workers, had greater sensitivity and negative predictive value than chest radiography for the diagnosis of pneumothorax in emergency department patients.

**Supplementary Information:**

The online version contains supplementary material available at 10.1186/s13089-025-00441-5.

## Background

In the emergency department (ED), clinicians have traditionally diagnosed pneumothorax (PTX) in patients presenting with suggestive symptoms using a chest X-ray (CXR). Commonly a confirmatory study e.g. computed-tomography scan (CT-scan) is performed, and CT is considered the gold standard. Bedside CXR examinations rarely quantify the size of the PTX, and a CT-scan may not be feasible due to the need for transportation outside of the department, an unstable patient, time constraints, and concern for radiation exposure [1]. Bedside ultrasound is an imaging tool commonly used in emergency medicine departments [2]. The user-friendly non-invasive design of the devices makes it attractive to medical professionals attuned to making time-sensitive and accurate diagnoses. Bedside ultrasound or point-of-care-ultrasound is performed by the Emergency Healthcare Workers, which includes EPs, Emergency Medecine Residents and medical students working in the ED, and allows immediate integration of the imaging interpretation into the diagnostic decision-making [3]. Chest ultrasound (CUS) can play a major role in the management of an ED patient presenting with a PTX. First described in 1987, CUS for the diagnosis of PTX in human patients has become a common ED ultrasound application [4, 5]. Although some meta-analyses demonstrated better performance—with higher sensitivity and similar specificity—of CUS compared to CXR for the diagnosis of PTX the studies did not specifically select CUS performed by Emergency Healthcare Workers [6, 7]. CUS examinations performed in the radiology department are not optimal since patients leave the ED resulting in delayed diagnosis and decision making. Additionally, this is unsafe and not ideal in overcrowded EDs, especially if the patient is unstable. The identification of a lung point on CUS not only confirms the presence of a PTX but may also allow for bedside estimation of its size, enabling real-time clinical decisions such as whether to place a chest tube immediately or proceed with a CT scan for further evaluation if the PTX appears small. To date, no meta-analysis has evaluated the characteristics of the ultrasound device, e.g. probe, the specialty of the clinician operator, and the patient's characteristics e.g. age, sex, and sampling method to assess the CUS accuracy and reliability of a PTX diagnosis. Thus, we performed a systematic review of the literature and a meta-analysis to assess the performance of Emergency Healthcare Workers performed CUS in the ED. Secondary aims were to evaluate the presumed influence of characteristics of CUS, patients and PTX.

## Methods

### Literature search

We performed a systematic review and meta-analysis of studies reporting a comparison CUS and CXR for the diagnosis of PTX with CT as the gold standard.

### Eligibility criteria

Studies had to assess the performance of CUS and CXR, both individually and compared to CT-scan as a gold standard in the diagnosis of PTX. Articles needed to report at least one of the following diagnostic performance criteria: area under the curve (AUC), sensitivity, or specificity, for both CUS and CXR. The mean and standard deviation (SD) had to be reported.

### Search strategy

**Fig. 1:**
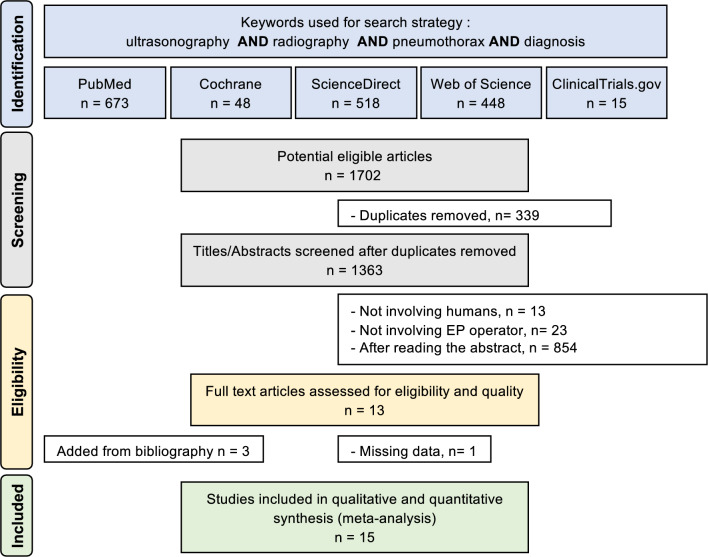
Search strategy

The search strategy is presented in Fig. 1. Using PRISMA guidelines, two authors (CB and JBBM) conducted the literature search, collated and reviewed the abstracts, and decided the suitability of the articles for inclusion. A third author (FD) reviewed any eligible article where the two researchers disagreed. We queried PubMed, Cochrane Library, ScienceDirect, ClinicalTrials.gov, and Web of Science databases from January 2000 through January 2024 using, the following search terms: “ultrasonography” OR “ultrasound” AND “chest radiography” OR “chest X-ray” AND “pneumothorax” AND “diagnosis”. Full research strategy is describe in the Appendix 1. No language restrictions were applied. All studies (retrospective and prospective) were included. We excluded studies performed in the pediatrics EDs.

### Data collection

For each study, the data collected was entered into an Excel^©^ sheet and included the first author’s name, publication year, country, study design, study aims, and outcome. The CUS characteristics included were type of probe, the probe frequency, the protocol utilized, inclusion and exclusion criteria, sampling method, population size, age, sex i.e. percentage of male, characteristics of the PTX (prevalence, type of trauma, size), the reference/gold standard for PTX in the population, the sensitivity and specificity with the standard deviation and the contingency tables.

### Risk of bias

**Fig. 2:**
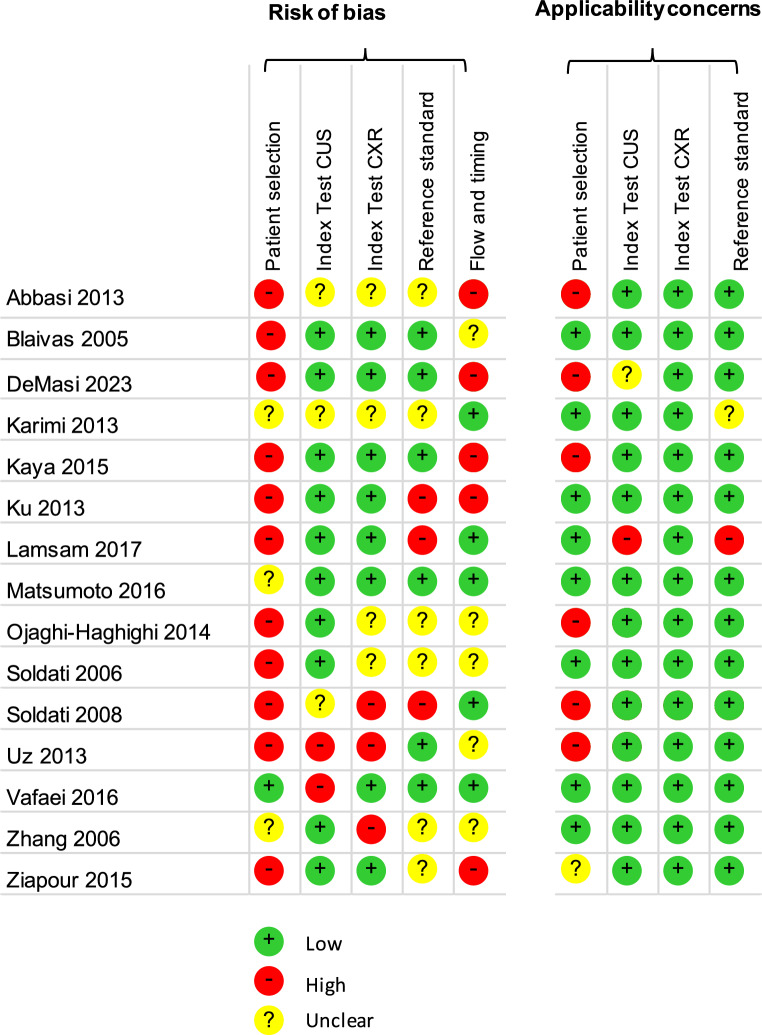
Risk of bias using the QUADAS-2 (Quality Assessment of Diagnostic Accuracy Studies) tool

Each study was assessed for sources by bias by using the QUADAS-2 (Quality Assessment of Diagnostic Accuracy Studies) tool [8]. The methodological areas analyzed are patient selection, interpretation of the tests (CUS and CXR), interpretation of the reference standard test (CT scan), and the time between CUS, CXR, and CT scan. The tool then assessed the external validity of the studies in three areas of applicability (patient selection, diagnostic examination, and reference test). A domain is typically rated as *low risk* if all signaling questions are answered favorably, and there are no concerns about study methods or conduct, *high risk* if one or more signaling questions are answered unfavorably, or if there’s a clear methodological flaw or *unclear risk* if there’s insufficient information to make a judgment. However, QUADAS-2 does not provide a standardized algorithm for combining these individual domain judgments into an overall risk of bias classification (e.g., high, moderate, or low) for the entire study. This synthesis is often left to the discretion of the reviewers (Fig. 2). The risk of bias for those nine items was defined as minimal, unclear, or high.

### Statistical considerations

Statistical analyses were conducted using Stata software (version 16, StataCorp, College Station, US). Main characteristics were synthesized for each study population and reported as the mean ± standard–deviation (SD) for continuous variables and the number (percentage—%) for categorical variables. We conducted random effects meta–analyses (DerSimonian and Laird approach [9]) on the performance on PTX diagnosis using sensitivity, specificity, positive predictive value (PPV) and negative predictive value (NPV). A Receiver Operating Characteristic (ROC) curve is generated by plotting (1-specificity) on the x-axis and sensitivity on the y-axis. For studies that reported only sensitivity and specificity, we calculated an overall summary AUC value by estimating a Summary ROC Curve (SROC) using the Metandi and Midas packages [10]. We evaluated heterogeneity in the study results by examining forest plots, confidence intervals (CI) and I-squared (I2). Formal tests for homogeneity based on the I2 statistic are the most common metric for measuring the magnitude of between–study heterogeneity and are easily interpretable. I2 values range from 0 to 100%, and are considered low for < 25%, modest for 25–50%, and high for > 50%. We further searched for potential publication bias using funnel plots of all aforementioned meta-analysis. We performed meta-regressions to compare performances of CUS and CXR. When possible (sufficient sample size), meta-regressions were also proposed to study the relationship between sensitivity, specificity, PPV, NPV and characteristics of population (age, gender), sampling method (convenience, consecutive), or characteristics of the ultrasonography (type of probe, frequency, US protocol performed) and PTX characteristics (prevalence, type of trauma). Results were expressed as regression coefficients and 95% CI. P values less than 0.05 were considered statistically significant. We did not prospectively registered this review.

## Results

An initial search resulted in 1702 articles (Fig. 1). The removal of duplicates and use of the selection criteria reduced the number of articles reporting a comparison between the diagnosis performance of CUS and CXR on PTX to twelve articles. In reviewing references of those twelve, we added three studies for a total of fifteen studies [11–25]. We performed the meta-analysis on these fifteen selected studies (Table 1).

**Table 1:** Characteristics of included studies

Study, year	Country	n	Sampling	Setting	Se US	Sp US	Se CXR	Sp CXR		Probe	US protocol	US sign
Abbasi et al. [11]	Iran	146	Convenience	Trauma	0.86	0.99	0.49		0.99	Linear	2th to 4th ICS at the hemi-clavicular lines 4th to 8th ICS at the mid axillar lines	LS, CT
Blaivas et al. [12]	Georgia	176	Convenience	Trauma	0.98	0.99	0.76		0.99	Convex	2th ICS at the hemi-clavicular lines 4th ICS at the antero-axillar lines	LS
DeMasi et al. [13]	United-States	846	Convenience	Trauma	0.21	0.99	0.24		0.99	Convex	E-FAST: no details	–
Karimi et al. [14]	Iran	140	Consecutive	Trauma	0.85	0.96	0.75		0.99	Linear	E-FAST: no details	LS, CT
Kaya et al. [15]	Turkey	212	Consecutive	Trauma	0.88	0.99	0.35		0.99	Linear	2th to 4th ICS at the hemi-clavicular lines 4th to 8th ICS at the mid axillar lines	LS, CT, BC
Ku et al. [16]	United-States	549	Convenience	Trauma	0.57	0.99	0.40		0.99	Convex	All ICS in hemi-clavicular lines	LS, CT, LP
Lamsam et al. [17]	United-States	59	Convenience	Respiratory	0.76	0.71	0.65		0.99	Linear	8 areas in each anterior and lateral region	LS, CT, LP
Matsumoto et al. [18]	Japan	159	Consecutive	Trauma	0.63	0.99	0.61		0.99	Convex	2th to 4th ICS at the hemi-clavicular lines 6th to 8th ICS at the mid axillar lines	LS, CT
Ojaghi-Haghighhi et al. [19]	Iran	150	Consecutive	Trauma	0.96	0.99	0.35		0.98	Linear	E-FAST: no details	LS, CT
Soldati et al. [20]	Italy	186	Consecutive	Trauma	0.98	0.99	0.54		0.99	Convex	3th ICS to down at the hemi-clavicular lines All ICS in para-sternal and mid axillar lines	LS, CT, LP
Soldati et al. [21]	Italy	109	Consecutive	Trauma	0.92	0.99	0.52		0.99	Convex	3th EIC to diaphragm at the hemi-clavicular lines All ICS in para-sternal and mid axillar lines	LS, CT, LP
Uz et al. [22]	Turkey	107	Convenience	Trauma	0.82	0.99	0.09		0.99	Both	4th or 5th ICS at the hemi-clavicular lines 4th or 5th ICS at the anterior axillar lines	LS, CT, BC
Vafaei et al. [23]	Iran	152	Consecutive	Trauma	0.84	0.98	0.67		0.93	Both	2th to 6th ICS in para-sternal, hemi-clavicular, anterior axillar et mid axillar lines	–
Zhang et al. [24]	China	135	Unclear	Trauma	0.86	0.97	0.28		0.99	Both	Each anterior, lateral and posterior region in supine position	LS, CT
Ziapour et al. [25]	Iran	45	Unclear	Trauma	0.78	0.92	0.36		0.99	Linear	3th ICS hemi-clavicular lines using two oblic incidences	LS, CT

*Se* Sensibility, *SP* Specificity, *US* Ultrasound, *CXR* Chest X-ray, *ICS* intercostal spaces, *FAST* Focused Assessment with Sonography in Trauma patient, *E-FAST* extented- Focused Assessment with Sonography in Trauma patient, *LS* lung sliding, *CT* comet tail artefact, *LP* lung point, *BC* Bar Code sign

### Risk of bias

Figure 2 presents the results of the risk of bias using the QUADAS-2 tool. The overall methodology quality was low. Regarding the patient selection domain, it can be explained by small sampling (low representability of the sample) and inappropriate exclusion criteria (hemodynamically unstable patients, chest wall injury precluding US exam). In some studies, the blinding methodology of outcome by operators interpreting CUS and CXR results was unclear. Radiologists may have had access to CT results when interpreting the CXR (Fig. 2).

### Study designs and objectives

Fourteen prospective studies and one retrospective [13] study were included in our analysis, performed between 2003 and 2023. Eight studies were carried out in Asia including five in Iran, four in Europe [12, 15, 20–22], and three in the United States [13, 16, 17]. All but two studies were single center in design [20, 21]. Two of the studies were not available in the English language [14, 22] so we enlisted the help of a collaborator (RB) for translation and data extraction. All the studies used CT as gold standard, alone or in parallel with chest tube placement. The chest tube was used as a reference when patients required emergent chest drainage before imaging tools e.g. concern for tension PTX.

### Recruitment, inclusion and exclusion criteria

The sequence ‘CXR then CUS’ or “CUS then CXR’ was defined by a non-random strategy, it was consecutive in seven studies, convenience in six [11–13, 16, 17, 22] and unclear for two studies [24, 25]. Five studies included patients regardless of age [14, 19, 23–25]. Eight excluded participants who were hemodynamically unstable or in which subcutaneous emphysema and/or probable tensions PTX was suspected [11, 15, 20–25]. Six excluded participants when a chest tube placement was done before any imaging tests [11, 13, 14, 16, 19, 21]. We excluded patients from our study when the articles did not provide descriptions of both the ultrasound examination and the imaging results.

### Population studied

*Sample size* Sample sizes ranged from 45 [25] to 846 patients [13]. We included a total of 3,171 patients and 702 PTX diagnosed.

*Age* Mean age was 40.2 years (95CI 30.1 to 50.2). This data was missing in three studies [17, 19, 25], one didn’t report the standard deviation [12].

*Gender* Men represented 71% (95CI 66 to 77) of the population analyzed, one study didn’t provide this data [25].

*Background* Our study comprised an analysis of 3112 trauma patients, among whom 1931 had sustained blunt chest trauma. Only a single study included patients exhibiting respiratory symptoms in the absence of trauma, accounting for 59 patients [17]. The primary causes of closed chest trauma were road traffic accidents, affecting 650 patients, followed by falls, which involved 266 patients. Additionally, 130 patients were identified with penetrating injuries.

### Pneumothorax

In our meta-analysis, the prevalence of PTX was 27.6% (95CI 20.9 to 34.3) of the total samples, ranging from 9 [16] to 51.4% [14].

*Size* Eight studies provided a classification of 483 pneumothoraxes according to size. Size was defined in the studies as the distance separating the thoracic pleura from the visceral pleural in the CT-scan analysis, with the following classification: large size (> 25 mm), medium size (between 10 and 25 mm), and small when < 10 mm. Of the PTX for which we had size measure, 32.1% were large (n = 155), 30.6% were medium (n = 148) and 37.3% were small (n = 180).

*Diagnostic signs* The absence of lung sliding, and comet tail artifact were the most frequently reported CUS findings [11, 14, 18, 19, 24, 25] for the diagnosis of PTX. Five studies included the lung point. Two studies described the use of the stratosphere sign in time motion ultrasound mode [15, 22]. [26, 33] One study [12] looked at lung sliding only (Table 1).

### Ultrasonography

*Operator* Emergency medicine resident performed the CUS in all but two studies. For one study, a medical student in the ED with a minimum training of two hours in ultrasound examination [17], participated in the CUS and the second paper included emergency medicine and traumatology operators, whose experience ranged from 38 to 258 ultrasound examinations [27]. No other demographic information of the operators was shared.

*Probe* The linear probe was used in six studies (752 patients, 204 PTX) [11, 14, 15, 17, 19, 25], whereas a convex probe was used in six studies (2,025 patients, 385 PTX) [12, 13, 16, 18, 20, 21], three studies used both (394 patients, 113 PTX) [22–24].

*Ultrasound protocol* The CUS scan protocol varied (Table 1). All but three studies utilized an extended CUS protocol i.e., more than four intercostal spaces (ICS) per hemithorax, anterior and lateral fields, following the mid-clavicular, anterior axillary or mid-axillary lines.

### Meta-analysis on the performance of pneumothorax diagnosis

**Fig. 3:**
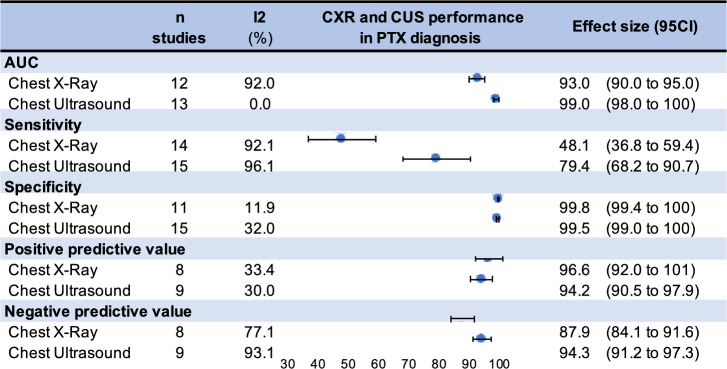
Summary of all meta-analyses for Chest X-Ray (CXR) and Chest Ultrasound (CUS) performance diagnosis in pneumothorax (PTX) for area under the curve (AUC), sensitivity, specificity, positive predictive value (PPV), negative predictive value. Blue dots represent the overall effect size for CXR and CUS in area under the curve (AUC), sensitivity, specificity, positive predictive value (PPV), negative predictive value. The lenght of each horizontal line around the dots represents their 95% confidence interval (95CI). I-squared (%): percentage of heterogeneity between studies for each meta-analysis

All meta-analyses on performance diagnosis of CUS and CXR are synthesized in Fig. 3. Specifically, sensitivity was 79.4%, 95CI 68.2 to 90.7% for CUS and 48.1%, 36.8 to 59.4% for CXR. The specificity of each imaging test was excellent and showed no difference, with 99.5%, 99 to 100% for CUS and 99.8%, 99.4% to 100% for CXR. The negative predictive value (NPV) was 94.3% (91.2 to 97.3%) versus 87.9%, 84.1 to 91.6% for CXR. However, positive predictive value was not significantly better for CXR (96.7%, 92 to 100%, 94.2%, 90.5 to 97.9% for CUS. The AUC of CUS obtained in the SROC curve was 99%, 98 to 100% and 93%, 90 to 95% for CXR. I squared (I2) was > 90% regarding the analysis of sensitivity describing a high degree of heterogeneity.

### Meta-regressions, comparing CUS vs CXR

**Fig. 4:**
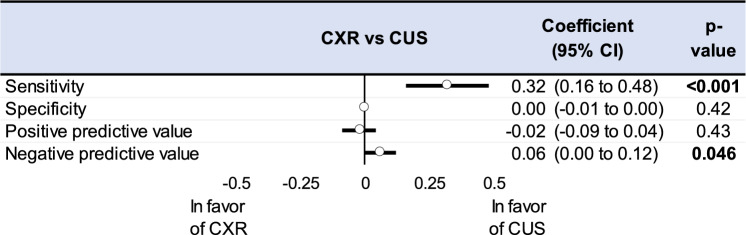
Summary of metaregressions on the performance of Chest X-Ray (CXR) and Chest Ultrasound (CUS) for sensitivity, specificity, positive predictive value (PPV), negative predictive value. The effect of each variable on the diagnosis performance is represented by a dot on a horizontal line in the forest-plot. The dots represent the coefficient for each variable, and the lenght of each line around the dots represent their 95% confidence interval (95CI). The black solid vertical line represents the null estimate (with a value of 0). Horizontal lines that cross the null vertical line represent non-significant difference between each examen

CUS had a better sensitivity than CXR (coefficient 0.31, 95CI 0.15 to 0.47). The accuracy of excluding PTX was better too for CUS through the negative predictive value (0.06, 0.001 to 0.12). It did not differ regarding the specificity (− 0.002, − 0.008 to 0.004) and positive predictive value (− 0.02, − 0.09 to 0.04) (Fig. 4).

### Meta-regressions, covariables

**Fig. 5:**
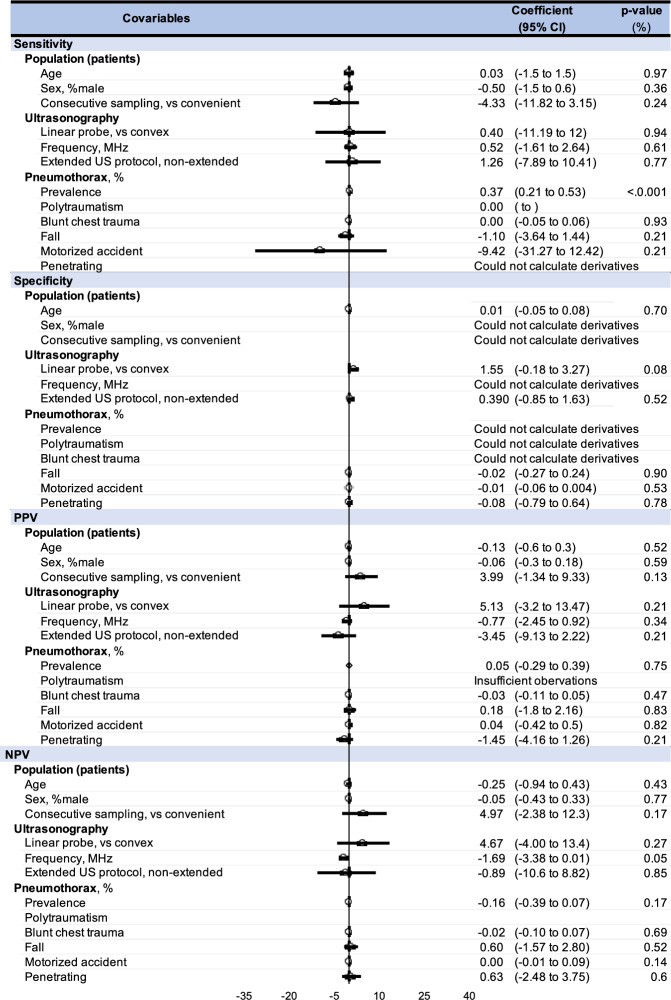
Summary of metaregressions i.e. variables influencing the performance of Chest X-Ray (CXR) and Chest Ultrasound (CUS) for sensitivity, specificity, positive predictive value (PPV), negative predictive value. The effect of each variable on the diagnosis performance is represented by a dot on a horizontal line in the forest-plot. The dots represent the coefficient for each variable, and the lenght of each line around the dots represent their 95% confidence interval (95CI). The black solid vertical line represents the null estimate (with a value of 0). Horizontal lines that cross the null vertical line represent non-significant difference between each examen (Fig. 6).

Our results showed that the performance of CUS and CXR was not related to sociodemographic characteristics (age, gender), probe characteristics (type of probe, frequency, or protocol), or PTX characteristics (prevalence, type of trauma) (Fig. 5). The meta-regressions did not show any influencing variables on the performance of CUS. Heterogeneity for the overall results of all aforementioned meta-analyses were high. Meta funnels confirmed the high heterogeneity with a lot of our studies outside of the base of the funnel, precluding further sensitivity analyses.

**Fig. 6:**
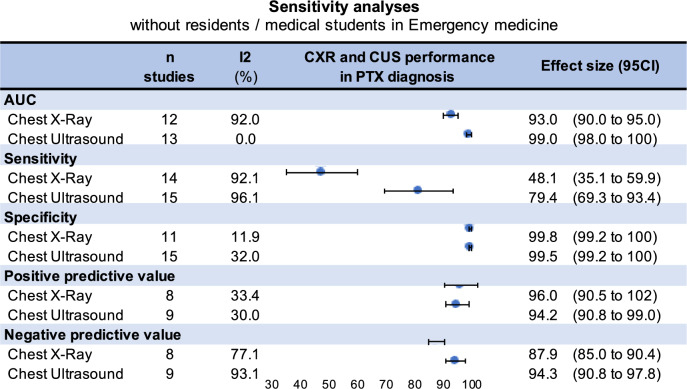
Sensitivity analysis excluding studies performed by residents or medical student

## Discussion

Our study assessed the performance of Emergency Healthcare Workers performed CUS for the diagnosis of patients with a PTX in the ED. This study reviewed the available literature for studies on the diagnostic accuracy of CUS compared to CXR for PTX. The studies were heterogeneous in their characteristics and results. The overall methodological quality was low due to insufficient information, especially in the patient selection domain. As a result, we encourage caution with the interpretation of the findings. 

### The performance of chest ultrasonography

CUS is better than CXR for the diagnosis of PTX. This finding is similar to those reported in the literature. Previously, two meta-analysis studies showed a greater sensitivity of CUS (88% and 87%, respectively) [6, 26]

With 79.4% sensitivity, our results were similar to those of Alrajab and colleagues (78.6%) [27], but different from others, such as Ding et al. [26] who analyzed 20 studies, but only four concerned EPs, or Alrajhi et al. [27] who included 18 studies, six of which were on EPs, and two studies were about iatrogenic PTX. One explanation for the difference in our results is that previous studies included intensivists, radiologists, surgeons, and EPs performing the CUS. The lowest sensitivity observed was in the sole retrospective study (21%) [13]. With an area under the SROC curve of 0.99, our results are comparable to those reported by Staub et al. (0.979) [7] (0.979) and Ebrahimi et al. [6] (0.99). In contrast, one retrospective study by Santorelli et al. showed that CXR had better performance than CUS in the diagnosis of PTX, with a false-negative rate for CUS of 36%, but this study had a retrospective design [28].

The absence of lung sliding, and comet tail artifacts was the most reported sonographic pattern for PTX in our studies. The combination of these signs has been shown to increase the diagnostic accuracy of PTX (sensitivity 100%, specificity 96.5%) [29]. The presence of a lung point is the most specific sonographic pattern of PTX, but its sensitivity is estimated at 60% [30]. The sensitivity of CXR may had been affected by the patient’s position. Almost all studies included in our review concerned trauma patients in which CXR was taken in a supine position, which can affect the ability to detect the air under the coastal margin. On this topic, some studies showed that other incidences such like oblique X-rays may increase the diagnosis of occult PTX [31].

### Ultrasonography in ED applications

Considering that CT is costly, time-consuming, and limited by the patient’s condition, a fast repeatable and cost-effective tool such as CUS may play an alternative role in the diagnosis of PTX [32, 33]. Some of the studies assessed the mean duration of the examination or time before diagnosis and showed that the use of CUS is faster and may help reduce the length of ED stay for patients [34]. It has been shown that CUS is not only effective in PTX diagnosis but also in other pulmonary diseases (haemothorax, pleural effusion, pneumonia) and abdominal injuries [35–38]. Being a radiation-free tool, it is more widely used in children and in pregnant women [39, 40]. In France, Bidault et al. noticed that between 2016 and 2023 the number of EDs equipped with US had increased from 74 to 88% and 28 to 69% for pre-hospital services [2]. Many advantages will allow this tool for primary survey in emergency settings, it could become useful in clinical bedside evaluation with the miniaturization of devices and improve ultrasound technology [41]. Ultrasound is helpful for clinicians in low-middle countries where advanced imaging modalities are often unavailable or in resource-limited settings in which ultrasound images can be acquired and transmitted on a telemedical platform for clinical diagnosis and management help from an expert interpreter [42]. Size evaluation and localization of PTX are essential for the proper management of the disease. Indeed, conservative management by observation or small catheters are the current standard of treatment. However, this data were not mentioned in the majority of the included articles, preventing us to study it.

## Limitations

We conducted this meta-analysis using published articles, so they are theoretically exposed to publication bias. Most studies were single-center thus, limiting the generalizability of the results. In addition, the geographical distribution showed the absence of countries in Africa, South America, Australia, and other regions making it impossible to extrapolate results to these populations. Included studies were heterogeneous according to funnel plots. This can be explained by high heterogeneous in ultrasonography characteristics (areas checked, type of probes, US machine). Moreover, the quality of operator training was not described in many of the studies, and the experience in US of each emergency healthcare worker was not clearly described. Inappropriate exclusion criteria were presented in most of our studies (hemodynamically unstable patient, exclusion when CUS was not performed because of chest wall injuries, patient excluded when missing one imaging exam). Hemodynamically unstable patients are likely those in whom CUS is most accurate, as they are unable to undergo CT scanning. In these cases, promptly diagnosing a PTX that requires immediate intervention is critical. The time between each examination may have influenced the results. CUS may had excluded the diagnosis of PTX during the primary survey but cause of a time delay before the CXR or the CT were performed., A small collection of air can grow and became more visible during the next imaging examination. Data on CXR were poorly describe and thus not included in this manuscript, which should be explicitly stated in the paper as it could act as a confounder to the results; for instance, if several CXRs were performed with patients in a supine position, this may explain the higher sensitivity observed for CUS in PTX detection, and results might differ if CXRs were performed with patients upright. Not all studies reported the characteristics of the population studied. Residual confounders might introduce some biases, for example, there was no information on the patient’s degree of severity, BMI, or vital signs. These can affect the performance of each imaging modality. Three studies presented the Injured Severity Score (ISS) and one gave vital signs data. The interpretation of CXR and CT were unclear regarding the blinding of the results of previous exam or clinical examinations. No studies but one assessed spontaneous PTX diagnosis by CUS. This study had lower sensitivity and specificity of CUS. Randomized clinical trials would better assess outcomes.

## Conclusion

In this meta-analysis, Emergency Healthcare Workers performed chest ultrasound was better than chest X-ray for the detection of a pneumothorax in emergency departments patients. Although included studies were subject to biases, especially in the selection of the population, these results could have great implications in pneumothorax detection and management in emergency departments. Future meta-analysis studies should evaluate the chest ultrasound diagnosis performance in spontaneous PTX.

## Supplementary Information

Supplementary material 1. Appendix 1. Detailed search strategy used within each database.

## Data Availability

All relevant data are within the paper. The datasets used and/or analysed during the current study are available from the corresponding author on reasonable request.
