# Supplementary material for: Chest ultrasound vs. Radiograph for pneumothorax diagnosis performed by emergency healthcare workers in the emergency department: a systematic review and meta-analysis
Source: Ultrasound J. 2025 Jul 31;17:37. doi: 10.1186/s13089-025-00441-5 (PMC12314293; doi:10.1186/s13089-025-00441-5)
Supplement: Supplementary file 1 — Supplementary material 1. Appendix 1. Detailed search strategy used within each database. [file 13089_2025_441_MOESM1_ESM.docx]

### Appendix 1. Detailed search strategy used within each database.

**PubMed**

(« Ultrasonography » OR « ultrasound » OR « sonography »)

AND (« Radiography » OR « x-ray » OR « x-rays » OR « CXR »)

AND (« Pneumothorax » OR « pneumothoraces » OR « hemopneumothorax »)

AND (« Diagnosis » OR « Diagnostic »)

Filter language: none

Filter date: range from 2000 to 2024

**Cochrane Library**

(« Ultrasonography » OR « ultrasound » OR « Sonography »)

AND (« Radiography » OR « x-ray » OR « x-rays » OR « CXR »)

AND (« Pneumothorax » OR « pneumothoraces » OR « hemopneumothorax »)

AND (« Diagnosis » OR « Diagnostic » OR « diagnosis accuracy »)

Filter date: range from 2000 to 2024

Filter language: none

**ScienceDirect**

(« ultrasound » OR « sonography ») **AND** (« radiography ») **AND** (« pneumothorax »)

**AND** (« diagnosis ») **AND** (« emergency department »)

Filter language: none

Filter date: range from 2000 to 2024

Filter article type: yes, selected review articles, research articles

Subject areas: yes, selected Medicine and Dentistry, Nursing and Health Professions

**ClinicalTrials.gov**

(« ultrasonography » OR « ultrasound »)

**AND** (« radiography » OR « x-ray » OR « x-rays » OR « CXR »)

**AND** (« diagnosis »)

**AND** (« Pneumothorax »)

Filter language: none

Filter date: range from 2000 to 2024

**Web Of Science**

(« Ultrasonography » OR « ultrasound » OR « Sonography »)

**AND** (« Radiography » OR « x-ray » OR « x-rays » OR « CXR »)

**AND** (« Pneumothorax » OR « pneumothoraces » OR « hemopneumothorax »)

**AND** (« Diagnosis » OR « Diagnostic » OR « diagnosis accuracy »)

Filter language: none

Filter dates: range from 2000 to 2024
